# The Effect of Egg Laying on Feather and Plasma Corticosterone Concentrations in Turkey (*Meleagris gallopavo*) Hens

**DOI:** 10.3390/ani11071892

**Published:** 2021-06-25

**Authors:** Emily M. Leishman, Nienke van Staaveren, Jeff Mohr, Benjamin J. Wood, Nikole E. Freeman, Amy E. M. Newman, Alexandra Harlander-Matauschek, Christine F. Baes

**Affiliations:** 1 Department of Animal Biosciences, Ontario Agricultural College, University of Guelph, Guelph, ON N1G 2W1, Canada; eleishma@uoguelph.ca (E.M.L.); nvanstaa@uoguelph.ca (N.v.S.); b.j.wood@uq.edu.au (B.J.W.); aharland@uoguelph.ca (A.H.-M.); 2 Hybrid Turkeys, Suite C, 650 Riverbend Drive, Kitchener, ON N2K 3S2, Canada; jeff.mohr@hendrix-genetics.com; 3 School of Veterinary Science, University of Queensland, Gatton, Queensland 4343, Australia; 4 Department of Integrative Biology, College of Biological Sciences, University of Guelph, Guelph, ON N1G 2W1, Canada; nfreeman@uoguelph.ca (N.E.F.); newman01@uoguelph.ca (A.E.M.N.); 5 Institute of Genetics, Vetsuisse Faculty, University of Bern, 3001 Bern, Switzerland

**Keywords:** glucocorticoid, breeding, avian, welfare, stress, poultry, biomarker

## Abstract

**Simple Summary:**

It is not known if an energetically demanding process, such as egg laying, can affect corticosterone measured in turkey feathers and blood plasma, or if there are differences between feather types and genetic lines. The objective of this study was to compare hormone levels from feathers and plasma taken before and during egg laying. We found that the corticosterone in the feathers were higher during egg laying, but only for one genetic line. This genetic line produces more eggs and, so, the elevated corticosterone may reflect the higher metabolic investment in producing eggs. Increases in corticosterone levels were found in different feather types; however, the overall hormone level differed between feather types. Unlike the feathers, we found a decrease in plasma hormone measures between the two periods which might reflect the influence of gonadal development or suppression of acute hormone production during lay. From this study, we conclude that feathers can be used to detect increases in corticosterone during periods of high energy demand, but the genetic line needs to be considered, and the results may not correspond with plasma measures. This study also reinforces the requirement for consistent feather sampling when using corticosterone levels in feathers as non-invasive biomarkers.

**Abstract:**

Phenological differences in energy demand (i.e., reproductive status) might influence the measurement of corticosterone. The objective of this study was to compare corticosterone concentrations in feathers (FCORT) and plasma (PCORT) for turkey hens before and during egg laying. Secondary feathers 1 and 3, and a plasma sample were collected from 50 hens at 30 weeks (before egg laying) from two purebred lines. The hens were reexamined during lay (45 weeks) to collect regrown feathers and plasma samples. Corticosterone concentrations were measured using an ELISA. Linear mixed models were used to assess the effect of genetic line (A vs. B) and period (pre-lay vs. lay) on FCORT and PCORT levels. An increase in FCORT during lay was detected for line B (*p* < 0.0001), but not line A (*p* = 0.3076). An increase in FCORT during lay was detectable in both feather types, but there was a difference between secondary 1 and 3 in FCORT concentration within each line studied. Conversely, PCORT decreased between the pre-lay and lay periods for both lines, although the decrease was more substantial for line A (*p* < 0.0001). Differences in metabolic investment in egg production between the two genetic lines may explain the different FCORT response during lay. The results from this study provide insight into how periods of high energy demand may influence corticosterone which should be considered when interpreting results.

## 1. Introduction

Corticosterone is the predominant avian glucocorticoid hormone which is an end-product of the hypothalamic-pituitary-adrenal (HPA) axis [1]. Corticosterone is often thought of in relation to its role in the adrenocortical response to perturbations where elevated levels act to mobilize energy, increase cardiovascular output, and shunt resources away from processes that are not critical for immediate survival (i.e., digestion, growth, reproduction) as part of a complex neural and endocrine response [1,2,3]. Aside from the role in the stress response, the primary role of glucocorticoids (like corticosterone) is the regulation of normal body functions and energy balance [2,3].

Glucocorticoid hormones, namely corticosterone, have been co-opted as indicators of stress or welfare in domestic birds [4]. Although measured differences in corticosterone may reflect HPA-axis activity in response to perturbations, it is often not considered, or known, how underlying factors might influence these measurements before we can make inferences about welfare [3]. Studies of physical or environmental perturbations often have a variable effect on corticosterone measurements which could possibly be attributed to differences in phenology, specifically underlying metabolic requirements which might influence glucocorticoid secretion [3,4,5]. To truly determine whether corticosterone provides an insight into animal welfare or stress, we need to determine how underlying metabolic demands might influence these processes.

A notable example of high metabolic demand is puberty and reproduction [6]. Since glucocorticoids play a key role in energy mobilization, we can expect elevated corticosterone levels during these times [7]. Corticosterone (measured in different tissue types) has been shown to be influenced by reproductive status in several species of domestic birds [8,9]. Corticosterone production is influenced by many aspects of the reproductive process such as sexual maturity and gonadal development [8] and egg laying [9]. Laying hen feathers that were collected at 28 weeks of age, after the onset of lay, contained significantly higher corticosterone concentrations compared to feathers collected at 16 weeks of age, before egg laying began [9]. Interestingly, several bird species have demonstrated the ability to dampen the adrenocortical response to acute stressors (i.e., restraint) when they are nesting or producing eggs [10,11,12]. Elevated corticosterone levels over the normal demands of reproduction typically have negative implications, so dampening the acute stress response may serve to maximize reproductive success ‘parental care hypothesis’, [13]. These findings may provide insight into the differing roles of corticosterone as part of energy balance (increasing during periods of high demand) and the stress response (dampened response during energetically demanding periods to prevent detrimental effects of elevated glucocorticoids on reproductive success).

These different roles of corticosterone (metabolic hormone vs. stress response) can potentially be captured by extracting hormones from tissues that offer different longitudinal perspectives on HPA-axis activity such as plasma (PCORT, minutes-hours) and feathers (FCORT, days-weeks). In more recent years, FCORT has been desirable because it is relatively non-invasive, retrospective, and a longitudinal record of corticosterone secretion [14]. The predominate route of corticosterone integration into feathers is believed to be during feather growth via diffusion from the blood quill [15]. This means that, when feather growth is complete, and the blood supply recedes, corticosterone is no longer internally deposited into the feather, although there may still be contributions from surface deposition of corticosterone (e.g., via preen oils) [15,16]. Since feather growth can take days–weeks depending on the species and/or feather type, this technique offers a different perspective compared to more acute measures. Although, during FCORT analysis, it is still worth considering concurrent plasma corticosterone (PCORT) levels. The relationship between FCORT and PCORT has been studied and, in general, basal measurements of PCORT are not correlated with FCORT [17,18,19]. However, PCORT measures after an acute perturbation (e.g., restraint) have been significantly correlated with FCORT from concurrently grown feathers [19,20,21]. Due to the difference in time representation between FCORT and PCORT, the measurement of one should not be used to infer the other, but both measures can provide insight into HPA-axis activity [19]. The relationship between PCORT and FCORT is unknown in domestic turkeys. Moreover, it is not well described how phenological differences, such as reproductive status, might influence these measurements, which is crucial to consider before they can be used as welfare indicators in poultry selection [22].

Although FCORT is now a widely used technique to study how poultry respond to their environment, there is an important methodological concern associated with its quantification. Specifically, there are demonstrated differences in the concentration of FCORT between different feathers collected from the same individual. In some cases, the intra-individual difference in FCORT can be significantly larger than the difference between individuals [23]. Significant differences between feathers can be found when the feathers are chosen from different body areas (i.e., back vs. tail feathers) [23,24]. Studies which assessed different feathers from the same area (i.e., primary feather 2 vs. primary feather 8) did not find a significant effect of feather type on FCORT levels [9,20]. This variability has not been well investigated in turkeys and is another important factor to consider when interpreting FCORT results.

The present study’s main objective was to assess the impact of egg laying on FCORT and PCORT in domestic turkeys in two purebred lines. It was hypothesized that, given the increased energetic demands of egg production, we would observe an increase in FCORT and PCORT in samples collected during the egg laying period. Secondary objectives were to examine the possible differences in FCORT levels from different feathers and examine the relationship between FCORT and PCORT.

## 2. Materials and Methods

### 2.1. Subjects and Experimental Design

**Figure 1:**
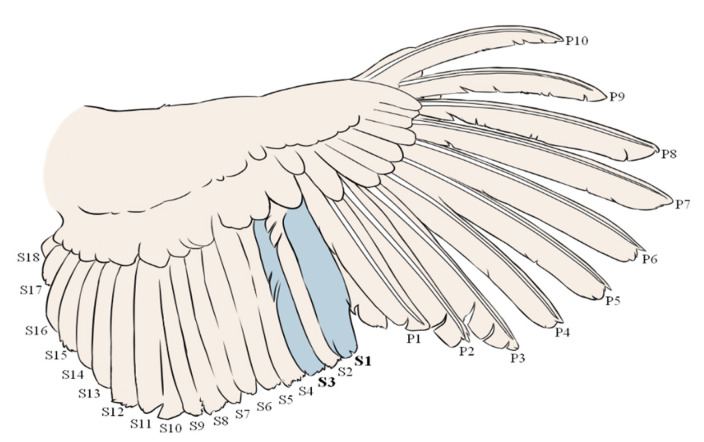
Schematic representation of the turkey wing feathers with numbering of the primary (P1–10) and secondary (S1–18) feathers. For FCORT analysis, secondary 1 (S1) and 3 (S3) were pulled at weeks 30 and 45. Illustration by Renée Garant.

Adult female turkeys (*Meleagris gallopavo*) from two different purebred lines (A and B) were randomly sampled from their respective flocks (housed within the same barn) for feathers and blood before egg laying at 30 weeks of age (hereafter ‘pre-lay’, A: 50 hens and B: 50 hens). Secondary 1 and 3 were plucked from the right wing of each hen by the same person (Figure 1). Plucking of secondary 1 and 3 allowed for easy identification and resampling of the regrown feathers when revisiting the flock 15 weeks later.

The two flocks (line A and B, respectively) were then reexamined at 45 weeks of age during the laying period (hereafter ‘lay’) to perform the second sampling on the same individuals. All birds were housed under standard conditions for parent stock hens, which were identical between the two genetic lines [25]. At week 30 (after the initial sampling), hens were transported from the rearing farm to the laying farm. In both the rearing and laying farm, wood shavings were used as bedding. Stocking density on the rearing and laying farms was approximately 3.5 sqft/bird and 6.0 sqft/bird, respectively. Diets and lighting regime were gradually changed based on the Hybrid Turkeys guidelines for parent stock hens [26,27]. Both genetic lines, on both the rearing and laying farm, were housed within the same barn and experienced the same changes in environmental and management conditions.

The genetic lines used in this study were selected for different breeding goals; the selection of line A is focused on production traits (i.e., body weight), and line B is focused on reproductive traits (i.e., egg production). All protocols complied with the guidelines of the Canadian Council on Animal Care and were approved by the University of Guelph Animal Care Committee (AUP 3782).

### 2.2. FCORT Extraction

The FCORT extraction protocol is based on methods described by Leishman et al. [22]. The whole feather was rinsed with water after collection to remove any dust/debris. Feathers were allowed to dry overnight and then stored in paper envelopes until extraction. During the extraction process, the whole feather (excluding rachis and calamus) was minced into pieces <5 mm^2^ and then ground using a bead mill with ceramic beads (Bead Blaster: Benchmark Scientific, Edison, NJ, USA). The resulting feather powder was weighed using an analytical balance (15 ± 0.1 mg, model accu-124D Dual Range, accuracy to 0.1 mg: Fisher Scientific, Toronto, ON, Canada) into a test tube. Methanol (5 mL, HPLC grade, Fisher Scientific) was added to each tube before placing in a sonicating water bath for 30 min. After sonicating, the tubes were moved to a shaking incubator at 50 °C for 12 h. Vacuum filtration with #4 Whatman filter paper was used to separate feather powder from methanol. During this process, the empty test tube was rinsed twice with 1 mL of additional methanol which was added to the extracted methanol (7 mL total). The methanol extract was evaporated at 40 °C under nitrogen gas using an evaporation plate. Extract residues were reconstituted with 500 µL of assay buffer immediately before the assay.

### 2.3. PCORT Extraction

On each occasion, blood samples were collected from approximately 10:00 a.m.–1:00 p.m. To confirm that time of day did not influence the PCORT concentrations, Pearson correlation coefficients were calculated for time of day and PCORT for each measurement period. For both periods, there was no significant correlation between time and PCORT (*p* > 0.05). The plasma processing and extraction protocol are based on kit manufacturer’s recommendations (mouse anti-rabbit IgG, Corticosterone ELISA kit, number 501,320, Cayman Chemicals, Cedarlane Labs, Burlington, ON, Canada). Blood samples were taken from the left brachial vein of each individual at each sampling period by the same trained person. Blood samples were taken within 2 min after restraint. Whole blood samples were centrifuged (Sorvall Legend RT centrifuge) at 1500× *g* for 20 min. Plasma was then separated and frozen at −20 °C until processing.

At processing, 125 μL of plasma was added to a clean test tube. Methylene chloride was added at 4× sample volume (500 μL, Fisher Scientific) and swirled to mix. Layers were allowed to separate, and then the upper methylene chloride layer was transferred to a clean vial. The washing with methylene chloride was repeated 4× for a total of 2 mL. The methylene chloride extracts from each sample were evaporated under nitrogen gas using an evaporation plate at 40 °C. Extract residues were reconstituted with 500 μL of assay buffer immediately before the assay.

### 2.4. Assay Evaluation and Procedure

A species pool for plasma (*n* = 10) and secondary feathers (*n* = 8) was created to determine the optimal sample mass for corticosterone extraction. Corticosterone was extracted from each pool and serial dilutions were created for the plasma (1500–11.7 μL) and secondary feathers (80–0.75 mg). Optimal sample mass was determined as the volume (μL) or mass (mg) that resulted in 50% binding for plasma (125 μL) and feathers (15.0 mg), respectively.

Samples were run across 16 ELISA plates (same as used for PCORT analysis) in duplicate. This assay kit has been previously validated for use with domestic turkey primary feathers [22]. Intra- and inter-assay coefficients of variation were 4.1% and 3.2%, respectively.

### 2.5. Statistical Methods

Linear mixed models were used to compare FCORT concentrations (response variable) between periods, genetic lines, and feathers using linear mixed models. Fixed effects included in the FCORT model were period (pre-lay or lay), genetic line (A or B), and feather (secondary 1 or secondary 3), and included all interactions. The repeated effect of period within the bird was included using the compound symmetry (cs) covariance structure. A logarithmic transformation was applied to the FCORT data to meet the assumption of normality and back-transformed with least-square means (LSmeans) presented. PCORT concentrations (response variable) were compared between periods and genetic lines using the same method but without the fixed effect of feather type. For both models, *p*-values for comparisons were adjusted using Tukey’s HSD. Pearson correlations between the FCORT values of secondary 1 and secondary 3, and PCORT, were calculated for each period (pre-lay and lay) separately.

To describe intra- and inter-individual variation in FCORT, standard deviation (SD, pg/mg) and coefficients of variation (CV, %) were calculated. Intra-individual variation was calculated as the difference between secondary 1 and 3 of each individual for both periods. The covariance parameter estimate of the FCORT model residuals was tested to determine if the variance is statistically different from zero (*p* < 0.05), indicating intra-individual variation after accounting for the model variables. Inter-individual variation was calculated for secondary 1 and 3 separately as the difference in FCORT concentration between individuals for the different feather types.

The α level for determination of significance was 0.05, and tendencies are reported between 0.05 and 0.10. All analyses were performed using SAS Studio (version 9.4, SAS Institute Inc., Cary, NC, USA).

## 3. Results

During the second sampling, it was not possible to locate all originally sampled individuals in the flocks. Additionally, both secondary 1 and secondary 3 did not regrow for some individuals, or were visibly damaged/broken, so only one feather may have been analyzed. Therefore, a final 73 birds were sampled for secondary 1, and 72 birds were sampled for secondary 3 (Table 1) during the laying period.

### 3.1. The Effect of Egg Laying on FCORT Is Dependent on Genetic Line

**Figure 2:**
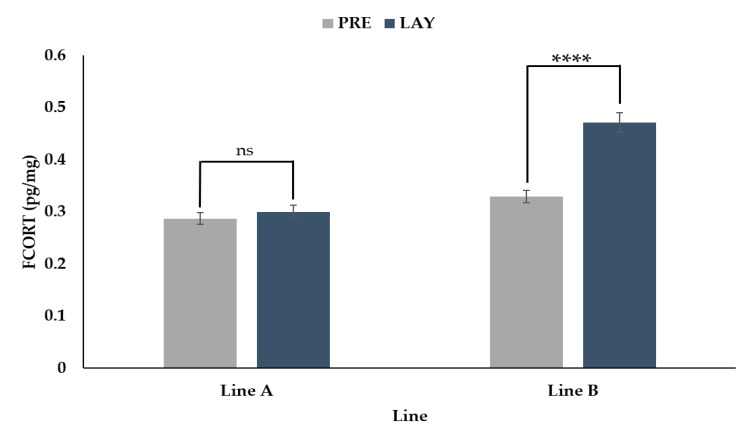
Back transformed LSMeans ± SEM for FCORT (pg/mg) for turkey hens from two different genetic lines (A and B) sampled before (PRE, 30 weeks of age) and during (LAY, 45 weeks of age) egg production (*n* = 73). *p*-values for simple effects denoted by ns = *p* > 0.05, * = *p* ≤ 0.05, ** = *p* ≤ 0.01, *** = *p* ≤ 0.001 and **** = *p* ≤ 0.0001.

The hens from the two genetic lines showed different responses in FCORT concentrations between the pre-lay and lay periods (Figure 2, *p* < 0.0001). For line A, there was no difference in FCORT between the pre-lay and lay periods (*p* = 0.3155). In contrast, birds in line B showed a 45% increase in FCORT from pre-lay to lay (*p* < 0.001).

### 3.2. Differences in FCORT between Feathers

**Figure 3:**
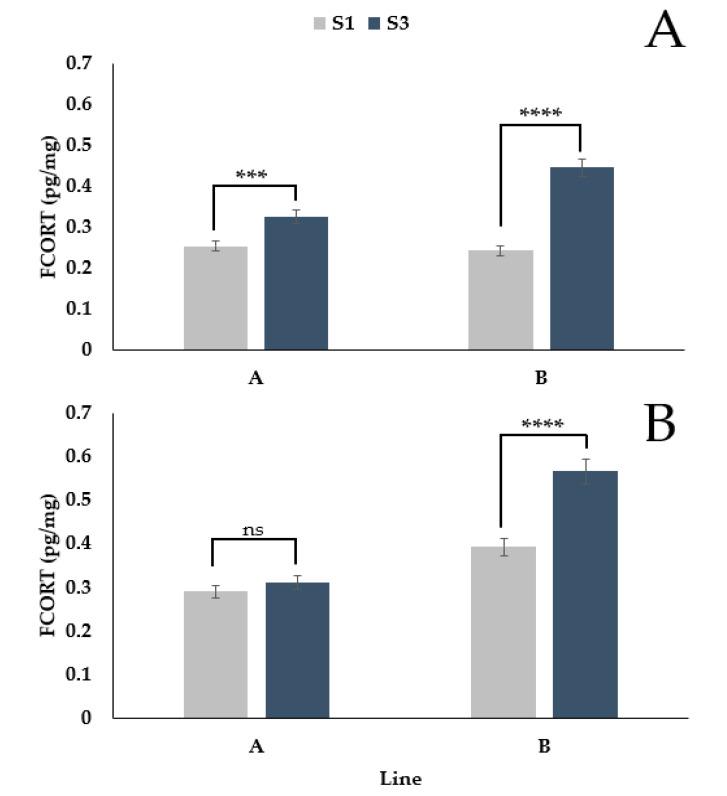
Back transformed LSMeans ± SEM for FCORT (pg/mg) from secondary feather 1 (S1) and secondary feather 3 (S3) for turkey hens from two different genetic lines during the pre-lay (**A**) and lay (**B**) periods (*n* = 73). *p*-values for simple effects denoted by ns = *p* > 0.05, * = *p* ≤ 0.05, ** = *p* ≤ 0.01, *** = *p* ≤ 0.001 and **** = *p* ≤ 0.0001.

There was a significant interaction between feather (secondary 1 vs. secondary 3) and genetic line (A vs. B) (Figure 3A,B, *p* < 0.0001). During the pre-lay period, FCORT levels of secondary 1 and secondary 3 differed in line A (*p* = 0.0001) and line B (*p* < 0.0001). During the laying period, there was no difference in the FCORT levels of the feathers for line A (*p* = 0.2832); however, there was a difference for line B (*p* < 0.0001). For both lines and periods, secondary 3 had higher FCORT levels than secondary 1. Still, the magnitude of the difference was greater for line B (pre-lay = 0.20 pg/mg; lay = 0.17 pg/mg) than line A (pre-lay = 0.07 pg/mg; lay = 0.02 pg/mg).

**Figure 4:**
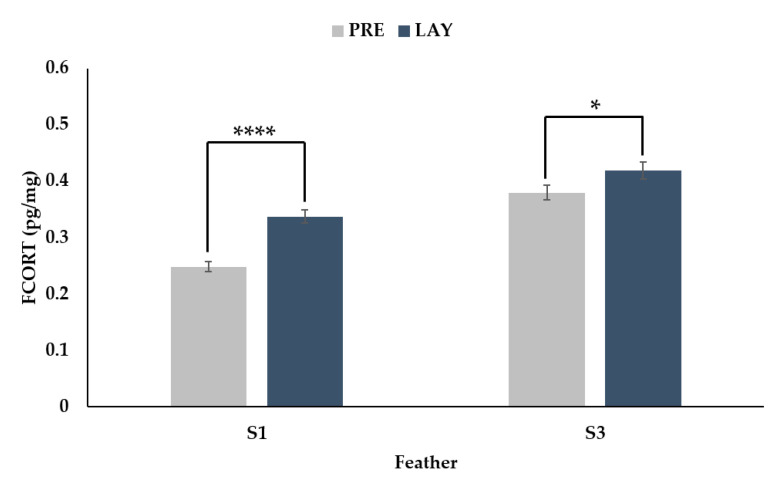
Back transformed LSMeans ± SEM for FCORT (pg/mg) from secondary feather 1 (S1) and secondary feather 3 (S3) sampled before (PRE, 30 weeks of age) and during (LAY, 45 weeks of age) egg production (*n* = 73). *p*-values for simple effects denoted by ns = *p* > 0.05, * = *p* ≤ 0.05, ** = *p* ≤ 0.01, *** = *p* ≤ 0.001 and **** = *p* ≤ 0.0001.

There was a significant interaction between feather (secondary 1 vs. secondary 3) and period (PRE vs. LAY) (Figure 4, *p* = 0.0011). For both feathers, the FCORT levels during lay were higher than pre-lay; however, the magnitude of the difference was greater for secondary 1 (36% increase, *p* < 0.0001) than secondary 3 (10% increase, *p* = 0.0281).

### 3.3. Intra- and Inter-Individual Variation in FCORT

Within an individual, the SD of FCORT concentration between the two secondary feathers ranged from 0–0.94 pg/mg across both periods, with an average SD of 0.12 pg/mg (Table 2). The CV ranged from 0–76% across both periods with an average of 29%. The results of the covtest indicated that the differences within an individual (variance of model residuals) were significantly different from zero (0.07 ± 0.007, 95%CI: 0.059–0.0879, *p* < 0.0001).

Between individuals, the average SD of FCORT concentration for secondary 1 was 0.17 pg/mg and 0.25 pg/mg for secondary 3 (Table 2). The average inter-individual CV was considerably higher (53–56%) than the intra-individual CV (29%) across both periods. However, there was much more variation between individuals during the lay period compared to the pre-lay period.

### 3.4. PCORT Decreases during Egg Laying

**Figure 5:**
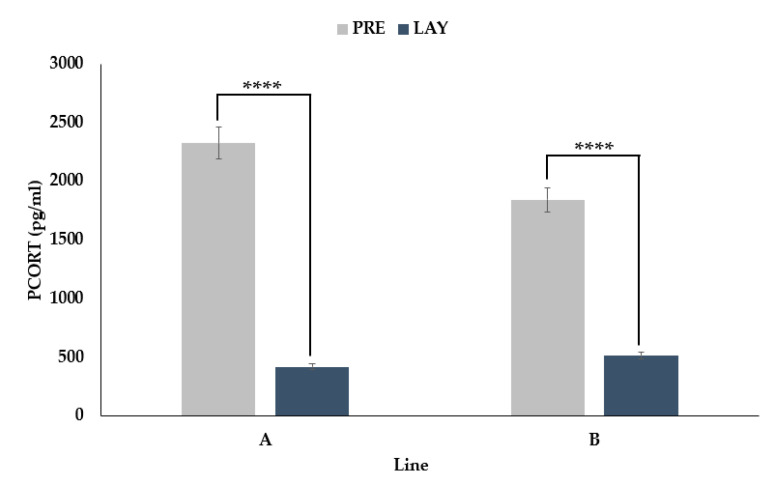
LSMeans ± SEM for PCORT (pg/mL) from two genetic lines (**A** and **B**) sampled before (PRE, 30 weeks of age) and during (LAY, 45 weeks of age) egg production (*n* = 73). *p*-values for simple effects denoted by ns = *p* > 0.05, * = *p* ≤ 0.05, ** = *p* ≤ 0.01, *** = *p* ≤ 0.001 and **** = *p* ≤ 0.0001.

An interaction between period and line was found (*p* < 0.0001) for PCORT. Conversely to the FCORT results, there was a decrease in PCORT for both line A (82% decrease, *p* < 0.0001) and line B (72% decrease, *p* < 0.0001) from the pre-lay to the lay period (Figure 5).

### 3.5. Correlations between FCORT and PCORT

The FCORT levels for secondary 1, secondary 3, and PCORT were not significantly correlated during the pre-lay period (Table 3). However, during the lay period, FCORT levels of secondary 1 and secondary 3 were positively correlated (*p* < 0.0001). Similarly, FCORT of both feathers was positively correlated with PCORT (*p* < 0.001), although the magnitude of the correlation with PCORT was smaller.

## 4. Discussion

This study aimed to investigate differences in FCORT and PCORT in two genetic lines (lines A and B) of domestic turkey hens before and during egg laying. A secondary objective was to compare the FCORT levels between different feathers (secondary 1 and secondary 3). We found that the effect of egg laying on FCORT was different for the two genetic lines, with an increase in FCORT during lay being found only for line B. We also found that the FCORT values for secondary 1 and secondary 3 were significantly different within each line. However, for both secondary 1 and secondary 3, there was a detectable increase in FCORT during the laying period. For PCORT, the magnitude of the effect of period was different for the two genetic lines. For both lines, PCORT decreased during lay, but the change was more substantial for line A.

The FCORT concentrations found for domestic turkey secondary feathers in this study (range = 0.15–2.04 pg/mg) are slightly lower than those reported in wild turkey secondary feathers (approx. 2.0–3.5 pg/mg); however, this may be due to differences in methodology (RIA vs. ELISA) as well as differences between wild and domestic birds [28]. Furthermore, Freeman and Newman [28] evaluated FCORT concentrations over a range of sample masses from 1–8 mg, whereas 15 mg was used in the present study, so these results may not be directly comparable. The same ELISA kit was also used by von Eugen et al. [29] and Nordquist et al. [9], who reported mean FCORT concentrations ranging from approximately 0.5–5.0 pg/mg and 1.5–3.0 pg/mg, respectively, for laying hen primary feathers. The FCORT values reported in our study are on the lower end of these reported ranges, but there is still some overlap. It is possible that the concentrations reported by these studies are higher because they were using primary feathers which have a larger blood supply compared to secondary feathers; therefore, greater internal deposition of FCORT can be expected [15]. Additionally, species-specific differences could have played a role as both of these studies used chickens (laying hens).

### 4.1. Effect of Egg Laying on FCORT and PCORT

For birds, the process of egg laying is energetically demanding [6]. Since glucocorticoids, like corticosterone, play a key role in energy mobilization [30], we expected to find higher levels of FCORT and PCORT in samples taken during the laying period.

In particular, for the feather samples, FCORT concentrations during the lay period would represent time-deposited corticosterone from the 14-week period in which the feather was regrowing. We found an increase in FCORT for the female-line B (stronger selection for reproduction), but no difference between the pre-lay and lay periods was detected for male-line A (stronger selection for growth traits). A previous study indicated that male-line and female-line turkey hens differ in reproductive development; female-line birds showed, e.g., earlier oviduct development [31]. The female-line (line B) produces more eggs than the male-line (line A) (Jeff Mohr, pers. comm.), suggesting that line B invests more resources in egg production compared to line A, which could explain why differences in FCORT were only observed in line B. Greater resource investment in egg production likely requires increased levels of glucocorticoids due to their role in energy mobilization [32]. This effect was seen in the study by Bortolotti et al. [14], who found that FCORT concentrations in primary wing feathers from red-legged partridges were highly and positively correlated to the number of eggs laid. Similarly, bird species with longer breeding seasons have lower baseline corticosterone levels compared to species with shorter breeding seasons likely because the intensity of reproductive effort is higher during shorter seasons thus requiring greater energy mobilization [33]. Unfortunately, we do not have individual production records for the birds involved in this study to corroborate these hypotheses, but it provides an interesting avenue for future research. It must be acknowledged that birds were transported from the rearing to the laying farm after the pre-lay measurement. Therefore, during egg laying, birds were also subject to changes in husbandry conditions, which may have influenced FCORT levels in addition to the demands of egg laying. However, these changes in husbandry conditions were identical for both lines. Given that there was no difference in FCORT detected for line A between the two periods while there was an increase in line B, this supports the hypothesis that some other factor besides housing and management influenced FCORT concentrations. Regardless, we cannot completely rule out that the genetic lines responded differently to the effects of housing and management factors given the experimental design. For example, both lines were fed the same diets with the same nutrient profile throughout the duration of the experiment [26]. Since we did not observe an increase in FCORT during the laying period for line A, this may indicate that the energy derived from the diet was sufficient to support the reproductive demand due to this line producing less eggs. Line B, due to its high rate of production, may require additional energy mobilization (i.e., elevated glucocorticoids) than what is provided in the diet which may explain the elevation in FCORT for line B only. In addition to further studies of FCORT and reproductive success, manipulation of the energy content of the diet should be considered as a potential driver of glucocorticoid levels during egg laying.

Glucocorticoids have been linked to the onset of sexual maturity in many species, including laying hens that showed increases in FCORT during egg production [9]. The elevations in glucocorticoids during egg laying are necessary to ensure sufficient energy to support high energetic demands during this time. However, if the animal is already in a negative energy balance or if it is exposed to other unpredictable stressors, corticosterone levels may be elevated over the normal demands of reproduction [34]. In these situations, the level of corticosterone can become detrimental to fitness by divesting resources away from “non-essential” functions such as growth, immunity, and reproduction [1,2,34]. This could explain why experimental treatment of birds with corticosterone delays the onset of lay and decreases egg production [35,36]. In our study, we did not use experimental manipulations of corticosterone concentrations to test how subjecting laying birds to additional perturbations influences corticosterone measurements. Although we did observe an increase in FCORT during lay in line B, it is likely that this is part of the predictable energetic demands of reproduction, as line B continued to have superior egg production results (Jeff Mohr, pers. comm.). It is possible that applying a perturbation to line B will further elevate corticosterone levels and have negative consequences for egg production. Future research is needed to elucidate which aspects of differences in investment in reproductive traits (e.g., oviduct development, egg number, age at first egg) are underlying the observed differences in FCORT between the female-line (B) and male-line (A) hens.

The relationship between egg laying and PCORT levels is less clear. A study of Japanese quail demonstrated that, in females, PCORT is significantly increased between 4 to 6 weeks of age (the period of gonadal development); however, the PCORT level dropped by nine weeks of age, and then even lower at 12 weeks of age [8]. This suggests that PCORT may be elevated during acute sexual maturity (gonadal development); however, PCORT continues to decrease after this time. This may explain why we saw a significant decrease in PCORT levels between our two time periods in both genetic lines. Since our plasma sample taken during lay was collected at 45 weeks of age, well into the egg production period, it is possible PCORT had already decreased. Moreover, since we took our first plasma sample at 30 weeks of age, soon before the onset of egg production, it may be more likely that we actually found elevated levels of PCORT as this may still be in the stage of gonadal maturation. Significant increases (>200%) in ovary and oviduct weight have been observed in female turkeys between 224–227 days of age (32 weeks of age) [31]. Assuming the reproductive development in our study (roughly 20 years later) is similar to this trajectory, our plasma sample during the pre-lay period would be closer to the time of gonadal development (and potentially elevated corticosterone), compared to our sample during the laying period. Acute increases in PCORT may also be suppressed during the breeding season to avoid the detrimental effects of perturbation-induced corticosterone levels on reproductive performance [11,37]. Adrenocortical modulation of the acute stress response has been demonstrated in a variety of species (albeit not well described in poultry) in birds with larger clutch sizes [10], greater rearing success [12], and nesting birds [38]. In flycatchers, Pereyra and Wingfield [38] found that the adrenal response to stress (measured via serial PCORT samples) was higher in birds before nesting compared to after the clutch had been laid. It is possible that, during egg production, the turkeys in the present study were modulating their adrenocortical response to acute stimuli (e.g., handling) in order to optimize their reproductive success which resulted in lower PCORT measurements. However, serial PCORT measurements during the pre-lay and lay periods, as well as measures of reproductive success, would be needed to test this hypothesis. Lastly, although blood samples for PCORT determination were taken within two min of restraint (to decrease the likelihood of restraint-induced increases in PCORT), it is possible that, over time, the turkeys became more habituated to human interaction and handling, resulting in lower PCORT. It may be beneficial for future studies to increase the frequency of the plasma samples to have a better idea of how circulating PCORT levels change with age as turkeys progress through sexual maturity and into egg production.

It should also be mentioned that the concentrations of PCORT measured in our study are lower than concentrations typically reported in the literature for turkeys. PCORT levels have been previously measured in ovariectomized turkey hens during the first laying season between 1.82 ng/mL and 7.14 ng/mL, depending on the time of day [39]. Between the two lines in our study, the mean PCORT concentration ranged from approx. 0.418–2.284 ng/mL, depending on the period. The lower PCORT measured in our study compared to Proudman [39] is potentially a reflection of the differences between ELISA and RIA, differences between housing or management in the different studies, or differences between genetic lines.

### 4.2. FCORT Variability

We found that FCORT levels were significantly different between secondary 1 and secondary 3 during each sampling period, with the exception of feathers from line A during the laying period.

Differences between feathers and feather types in terms of FCORT have been reported previously [23]; however, this is not always the case. Others found that feathers of the same type tend to have similar FCORT levels (i.e., secondary 1 and secondary 2) [9,20], whereas feathers from different body areas (i.e., back feathers and wing feathers) tend to show differences [23,40]. This is potentially because FCORT deposition in feathers can be affected by the feather’s size, shape, colour, structure, and growth rate [19,40,41]. Feathers of a similar location are likely morphologically more similar and should have less variation in CORT deposition than a different feather type [15,40]. This is in contrast to our finding that secondary 3 typically had higher FCORT concentrations than secondary 1. However, since FCORT is deposited only during the period of feather growth, it is only valid to compare FCORT levels between feathers that have grown concurrently [40,42]. The natural growth pattern and molt of the wing feathers is staggered [42,43], and so secondary 1 and secondary 3 would not likely start and stop growing at the exact same time which may explain why we saw differences between the feathers during the pre-lay period. Based on an early description of turkey feather growth, post-juvenal secondary 1 grows between 20–25 weeks of age, whereas secondary 3 grows from 7–12 weeks of age and so the timing of growth of these two feathers before the first sampling (30 weeks) is quite different although the duration of growth is the same (5 weeks) [43]. However, when we performed our sampling, we plucked both feathers at the same time and initiated simultaneous regrowth, meaning that the period of hormone deposition during the lay period should be more closely aligned compared to the pre-lay period. Based on our observations, at 45 weeks, both feathers were mature to the point of no blood supply indicating their growing period was likely between 30–35 weeks. This hypothesis is supported by finding no difference in the FCORT concentrations of the two feathers for line A during egg laying. Moreover, this could explain why we did not find a correlation between secondary 1 and secondary 3 grown in the pre-lay period, but we did find a positive correlation between the two feathers in the lay period. However, contrary to this hypothesis, there was still a difference between the feathers of line B during the laying period. The FCORT levels of line B were more affected by egg laying than line A. Additionally, as suggested by Jenni-Eiermann et al. [15], this feather may have a larger blood supply that allows for more deposition of FCORT, especially during periods of elevated concentrations. This combination may explain the differences between the feathers for line B during egg laying. There is also some debate about the contributions of externally deposited corticosterone to FCORT measurements. Since corticosterone, a steroid hormone, is lipophilic, it was initially suggested by Bortolotti et al. [14] that it may be transferred onto feathers via preen oils. There is some evidence to support this hypothesis such as a reduction in FCORT when feathers are washed with detergent prior to analysis [15] or an increase in FCORT concentrations in mature feathers [9,16]. However, other studies have found no difference in FCORT concentrations after washing feathers [14] or were unable to find evidence of corticosterone in preen oils [20]. If corticosterone is transferred in preen oils by preening behavior, it is possible that this may explain some of the variation in FCORT concentrations observed between feathers as all feathers are likely not preened equally. As we did not wash feathers with a detergent (water only) prior to extraction, there may be the potential for external deposition of corticosterone to influence some of the variability between and within individuals observed in this study. Further work should concentrate on this hypothesis and the relative importance of internal versus external FCORT deposition. Additionally, it should be investigated how differences in feather preparation before extraction (washing with water vs. detergent) may influence results [19].

Aside from the quantitative differences in FCORT concentrations between the feathers, we could still discern an increase in FCORT during the laying period in both secondary 1 and secondary 3. While the current study only used secondary feathers, and did not sample other feathers (e.g., back or tail feathers), we cannot state for certain that differences in FCORT during lay would be found in all feather types. The differences in FCORT value between the feathers, even of a similar type, emphasize the need for consistency in sampling.

Even though the FCORT concentrations of secondary 1 and 3 were significantly different within the studied lines, we found that the inter-individual variation in FCORT was larger than the intra-individual variation when looking at both periods (53–56% vs. 29%). This is contrary to what was found in chickens by Häffelin et al. [23], who found that intra-individual variation was higher than the variation between birds. However, they analyzed many more feather types (scapular, tail, wing, e.tc.) than the present study. Furthermore, the feathers were collected at the end of the rearing period (before egg production) so the values from their study may better represent the “pre-lay” variation in FCORT. This may explain why, in the present study, the intra-individual variation (32%) is slightly greater than the variation between individuals (25–30%) during the pre-lay period since this is supposed to reflect levels of FCORT before egg production. Inter-individual variation is an important consideration when assessing how individuals respond to the same situation [23]. Our results indicate more variability in the FCORT response between birds than within birds during energetically demanding periods, which may relate to differences found between genetic lines. Aside from genetic lines, some of this variability may also be explained by individual differences in feather wear (e.g., abrasions or barbule breakage), which is expected to influence FCORT concentrations due to potential loss of feather material, and thus corticosterone [19]. This can introduce some variation as all individuals or all feathers within an individual are likely not affected equally. Feather damage is an issue of particular relevance for domestic poultry because of problematic behaviors like injurious pecking [44] or the development of feather deformities such as fault bars [45]. While we chose not to analyze samples which were visibly damaged/broken, it is possible that variation in feather wear influenced the variability in FCORT seen between the studied variables. The prevalence and impact of feather quality and fault bars on FCORT concentrations in turkeys has yet to be assessed but is an important consideration to better understand FCORT variability. Still, the present results provide an important benchmark for FCORT variability within and between individual turkeys, which is not well described in the literature.

### 4.3. Relationship between FCORT and PCORT

Several studies have demonstrated strong correlations between experimentally elevated plasma CORT and concentration of CORT in feathers [19,20]. These studies include the use of corticosterone-filled silastic implants [20], corticosterone time-release pellets [21], or dexamethasone, an inhibitor of corticosterone deposition [46]. However, plasma CORT measures are typically in the nanogram range, whereas FCORT is typically in the picogram range, which indicates that the relationship is not one-to-one [19]. We did not find a significant correlation between PCORT and FCORT from secondary 1 and secondary 3 during pre-lay; however, there was a significant correlation between PCORT and FCORT during egg laying. This is similar to the findings of Bortolotti et al. [14], who did not find a significant correlation between FCORT and PCORT during the control period, but did find a significant correlation during a stress-induced treatment period. However, it should not be expected that PCORT correlates highly to FCORT [17,18,19]. Plasma titers represent a brief snapshot of CORT (minutes to hours), sensitive to circadian rhythm, which may not align relevantly with a long-term integrated measure obtained from feathers (days-weeks) [19]. Due to these differences, FCORT should not be used to infer plasma levels but be used as a complementary measure [19].

## 5. Conclusions

The aim of this study was to compare the FCORT concentrations from domestic turkey hens before and during egg laying using two different genetic lines and two different feathers. The effect of egg laying on FCORT was significantly influenced by genetic line, with increases in FCORT during egg laying only being observed in the line selected for reproductive traits. The results of this study demonstrate that understanding individual variation in life-history investment may help explain the large amount of variability seen in studies of FCORT and PCORT. Furthermore, an increase in FCORT during egg production was detectable for both secondary feathers, though differences between the feathers for both genetic lines existed. These results emphasize the importance of consistency in feather sampling when assessing biomarkers such as corticosterone. Conversely to the FCORT results, we found a significant decrease in PCORT levels during egg production, potentially due to the pre-lay sampling reflecting elevated levels from reproductive development or modulation of acute HPA-axis activity during egg laying. Future studies should focus on collecting detailed reproduction parameters and FCORT measurements to determine which differences in reproductive investment between the genetic lines contributed to the observed FCORT differences. This study also highlights that, for FCORT to be used as a biomarker energy balance, or to make inferences about chronic stress, there is a need for clear understanding of physiological processes that can influence the measured concentrations.

## Figures and Tables

**Table 1:** Number of birds (N_birds_) with secondary 1, secondary 3, and plasma samples collected during the pre-lay (30 weeks of age) and laying periods (45 weeks of age). Birds which could not be relocated during egg laying were excluded from the analysis.

Sample Type	Total	Line A	Line B
Secondary 1	73	37	36
Secondary 3	72	34	38
Plasma	73	37	36

**Table 2:** Intra- and inter-individual standard deviation (SD, pg/mg) and coefficient of variation (CV, %) in FCORT concentration during the pre-lay period (PRE, 30 weeks of age), laying period (LAY; 45 weeks of age), and both periods combined (BOTH). Intra-individual variation represents difference in FCORT between secondary 1 (S1) and 3 (S3) from the same individual. Inter-individual variation represents difference in S1 and S3 FCORT between individuals.

Period	Intra-Individual		Inter-Individual (S1)		Inter-Individual (S3)	
	SD	CV	SD	CV	SD	CV
PRE	0.11	32.58	0.07	25.41	0.12	29.96
LAY	0.14	26.15	0.22	55.22	0.33	64.91
BOTH	0.12	29.37	0.17	53.48	0.25	55.75

**Table 3:** Pearson correlation coefficients between secondary 1 FCORT (FCORT_S1), secondary 3 FCORT (FCORT_S3), and PCORT during the pre-lay period (above diagonal) and laying period (below diagonal) (*n* = 73).

Variable	FCORT_S1	FCORT_S3	PCORT
FCORT_S1		0.15	0.13
FCORT_S3	0.57 ^1^		−0.15
PCORT	0.50 ^1^	0.43 ^2^	

^1^ *p* < 0.0001. ^2^ *p* < 0.001.

## Data Availability

Data available from the corresponding author upon reasonable request.
